# Stimbiotic supplementation modulated intestinal inflammatory response and improved boilers performance in an experimentally-induced necrotic enteritis infection model

**DOI:** 10.1186/s40104-022-00753-9

**Published:** 2022-09-14

**Authors:** Ji Hwan Lee, Byongkon Lee, Xavière Rousseau, Gilson A. Gomes, Han Jin Oh, Yong Ju Kim, Se Yeon Chang, Jae Woo An, Young Bin Go, Dong Cheol Song, Hyun Ah Cho, Jin Ho Cho

**Affiliations:** 1 Department of Animal Science, Chungbuk National University, Cheongju, 28644 South Korea; 2 Cherrybro Co., Ltd., Jincheon-Gun, 27820 South Korea; 3 AB Vista, Marlborough, Wiltshire UK

**Keywords:** Broiler chicken, *Clostridium perfringens*, Necrotic enteritis, Xylanse, Xylooligosaccharide

## Abstract

**Background:**

Two experiments were conducted to establish an optimal NE challenge model and evaluate the efficacy of stimbiotic (STB) supplementation in necrotic enteritis (NE) challenged broilers. In Exp. 1, a total of 120 Arbor Acres (AA) broilers (45.0 ± 0.21 g) were randomly assigned to 6 treatments in a 3 × 2 factorial arrangement. Vaccine treatments included non-challenge (0), × 10 the recommended dose (× 10) or × 20 the recommended dose (× 20) by the manufacturer. *Clostridium perfringens* (CP) treatments were non-challenge (No) or 3 mL of 2.2 × 10^7^ CFU CP challenge (Yes). In Exp. 2, a total of 72 AA broilers (40.17 ± 0.27 g) were randomly assigned to 6 treatments in a 3 × 2 factorial arrangement. Dietary treatments included non-additive (CON), 100 mg/kg STB (STB) and 100 mg/kg STB on top of a typical commercial blend including an essential oil, probiotics, and enzyme (CB). Challenge treatments included non-NE challenge (No) and NE challenge (Yes) as established in Exp. 1.

**Results:**

In Exp. 1, CP and vaccine challenge decreased (*P* < 0.05) body weight (BW), body weight gain (BWG) and feed intake (FI), and increased (*P* < 0.05) the number of broilers with diarrhea and intestinal lesions. The oral administration of × 20 recommended dose of vaccines coupled with 3 mL of 2.2 × 10^7^ CFU CP resulted in (*P* < 0.01) a significantly increased incidence of wet litter and intestinal lesions. Thus, this treatment was chosen as the challenge model for the successful inducement of NE in Exp. 2. In Exp. 2, the NE challenge negatively affected (*P* < 0.01) growth performance, ileal morphology, immunoglobulin contents in blood, caecal microbiota in the caecum, footpad dermatitis, intestinal lesion scores, tumour necrosis factor (*TNF-α*) and endotoxin in the serum compared with the non-NE challenged birds. The supplementation of STB and CB in diets enhanced (*P* < 0.05) growth performance, intestinal microbiota, and blood profiles by stimulating ileal morphology (VH and VH:CD) and propionate production in the cecum, and there were no differences in measured variables between STB and CB supplemented birds.

**Conclusion:**

Overall, these results indicate that STB supplementation was able to reduce the inflammatory response and improve the performance of NE challenged birds, and the supplementation of STB alone was as effective as a typical commercial blend containing a number of other additives.

## Background

Necrotic enteritis (NE), caused by *Clostridium perfringens* (CP) and exacerbated when birds are co-infected with Eimeria spp., is one of the most severe and common disease resulting from intestinal mucosal damage [1–3]. NE occurs between 2 and 6 weeks of age and leads to increased intestinal damage and mortality rate in broilers [4–7] with symptoms such as melena, decreased growth performance, depression, and diarrhea [4, 8]. Antibiotic growth promoters (AGP) have long been used in poultry feed to control enteric diseases, including NE [1, 9]. However, the use of antibiotics and antimicrobial growth promoters in livestock production has been banned in recent years [10], leading to increased NE incidence, imbalance of gut microflora and decreased nutrient digestibility in broilers [11]. As a result, many researchers have been searching for alternative methods and strategies to enhance the host’s health by manipulating the intestinal microbiota and improving immunity. Among these alternatives, probiotics, prebiotics, organic acids, and phytogenic extracts have shown some potential [12–14]. Poultry diets often contain considerable levels of arabinoxylans as part of non-starch polysaccharides from grains and plant protein meals. Xylanase in poultry diets has become indispensable to optimize nutrient utilization [15]. Xylanase is a carbohydrase that hydrolyzes the β-(1–4) glycosidic bonds of arabinoxylans, and thus, has the potential to partially reduce the anti-nutritive effects of dietary fibre [16]. Xylo-oligosaccharides (XOS) produced by exogenous xylanase (EX) acts either directly as a prebiotic or a signalling molecule to stimulate fibre-degrading microbiota and cross-feeding mechanisms [17]. However, a broiler study reported that the amount of XOS generated by EX is somewhat dependent on the structure of the fibre in the basal diet and often is not sufficient to explain the increased butyrate and other short-chain fatty acids (SCFA) in the caeca [18]. Ribeiro et al. [19] stated that a small level of XOS is more likely to perform as an activator of specific bacteria in the gastrointestinal tract (GIT) than directly act as a quantitative prebiotic. The stimbiotic (STB) concept has been recently introduced as a non-digestible and fermentable additive that stimulates the development of a proportion of the microbiome comprising bacterial species that are principally involved in fibre degradation [17, 20]. It was reported that a low concentration of STB induced a better fermentation activity in the colonic samples compared with 10–20 times higher doses of commercially available prebiotics. To successfully test the proposed mode of action, it is critical to develop an experimental infection model that will mimic a commercial outbreak of NE. Many researchers tried to study the impact of additives on the severity of NE disease but often failed in developing the disease [21, 22]. Therefore, for successful NE induction in the second study, we performed a pre-screening experiment (Exp. 1) to determine the optimal dosage of vaccine (coccidia and infectious bursal disease) and CP for inducing damage of the intestinal mucosa and instigating an NE infection model. We then conducted the second experiment to determine the effects of STB on performance and intestinal barrier function in broilers challenged with the NE infection model. The hypothesis tested in Exp. 1 was that the increased dose of the commercial cocci and IBD vaccines to suppress the immune response prior to the oral dose of CP will increase the severity of NE. The hypotheses tested in Exp. 2 were that; (1) experimental induction of NE will increase inflammatory response and reduce the performance of broilers, (2) supplementation of STB will partly reduce the extent of the performance loss and the inflammatory response, and (3) supplementation of additives with the STB will show a limited additional beneficial effect in broilers challenged with NE.

## Material and methods

### Exp. 1

#### Oral administration of vaccine and *C. perfringens* (CP)

We used two coccidia vaccine (Hipra Evalon®, Laboratorios Hipra, Girona, Spain). This vaccine composition is as follows: oocysts from of precocious lines of *Eimeria acervuline* (Strain 003, 332–450 oocysts per dose), *Eimeria brunetti* (Strain 034, 213–288 oocysts per dose), *Eimeria maxima* (Strain 013, 196–265 oocysts per dose), *Eimeria necatrix* (Strain 033, 340–460 oocysts per dose) and *Eimeria tenella* (Strain 004, 276–374 oocysts per dose). Infectious bursal disease (IBD) vaccine (IBD Blen®, Boehringer Ingelheim Animal Health USA Inc., Georgia, USA) were used at the same time. The freeze dried live intermediate strain of infectious bursal disease virus (IBDV) is as follows: infectious bursal disease Winterfield 2512. These vaccines were diluted in sterile water and 10 times or 20 times the recommended dose was administered orally through a sterile pipette (BNF-TIP-1000, BNF Korea, China) at 14 days of age. Uninfected control broilers received the same dosage of sterile phosphate-buffered saline via oral gavage. Feed was withdrawn for 6 h and water was withdrawn for 12 h prior to administration of vaccines. CP type A NCTC 8798 (NCTC, National Collection of Type Cultures, London, UK) were used in this experiment. In 4 d after vaccination, a total of 3 mL of CP at 2.2 × 10^7^ colony forming units (CFU) was orally administered twice daily for 3 d (09:00 and 17:00; on 18, 19, and 20 d).

##### Experimental animals and treatment

One-hundred and twenty 1-day-old (initial body weight: 45.0 ± 0.2 g) Arbor Acres broilers were obtained from a local hatchery (Cherrybro Co., Asan, Korea) and randomly allocated to a 3 × 2 factorial arrangement. Each treatment consisted of 5 replicate cages with four broilers per cage. Cage and plastic plate to collect the fecal sample for analyzing the incidence of diarrhea were divided into four-space. Broiler chickens were raised in the same feed and environment until 14 days of age, and the environment of the room was maintained at 23 ± 1 ℃ and 50 ± 5% of relative humidity. The treatments were three doses of vaccines (0, 10 or 20 times the recommended dose) without and with oral CP challenge. The Hipra Evalon and IBD Blen® vaccines were administered via oral to immunocompromise the broiler chicken, and then the broilers were challenged with CP via oral gavaging 3 mL with 2.2 × 10^7^ CFU CP/mL for 3 d. Broiler chickens were offered starter, and grower diets from 1 to 14, and 14 to 23 d, respectively. Broiler chickens were fed with identical diets that were formulated to meet or exceed the NRC requirements (Table 1) [23] without antimicrobial product throughout the 24 d experimental period. All broiler chickens are allowed to consume feed and water ad libitum.

**Table 1:** Composition of the basal diets

Item, %	Pre-starter, 1–7 d	Starter, 7–14 d	Grower, 14–21 d	Finisher, 21–30 d
Corn grain	41.09	45.72	49.46	49.61
Wheat	12.00	12.00	12.00	12.00
Rice pollards	2.40	2.50	2.50	2.50
Soybean meal (45%)	26.05	21.58	18.06	13.27
Sesame meal (47%)	1.00	2.00	2.00	3.00
DDGS (28%)	6.00	6.00	5.00	8.81
Animal proteins (50%)	6.57	5.11	5.60	5.48
Animal fats	1.70	2.00	2.10	2.30
*L*-Lysine (55%)	0.65	0.67	0.65	0.66
*DL*-Methionine (88%)	0.38	0.34	0.35	0.35
*L*-Threonine (98%)	0.16	0.15	0.14	0.13
*L*-Tryptophan (98%)	0.01	0.03	0.10	0.10
Salt-proc (Fine)	0.21	0.20	0.20	0.21
Limestone	1.28	1.22	1.41	1.13
Mineral premix^1^	0.20	0.20	0.20	0.20
Vitamin premix^2^	0.13	0.11	0.10	0.10
Choline, 50%	0.10	0.10	0.08	0.08
Phytase1000	0.09	0.07	0.06	0.07
Calculated value, %				
AME, kcal/kg	3018	3030	3080	3110
Moisture	11.804	11.949	11.878	11.777
C Protein	23.402	21.155	20.112	19.508
Lysine	1.481	1.323	1.234	1.149
Methionine	0.739	0.664	0.667	0.674
TSAA	1.116	1.019	1.021	1.015
Threonine	0.996	0.909	0.857	0.809
Tryptophan	0.252	0.223	0.214	0.203
C Fat	5.539	5.824	6.079	6.515
C Fiber	3.151	3.121	2.943	3.145
C Ash	5.727	5.325	5.087	4.893
Calcium	0.850	0.800	0.750	0.700
Phosphorus	0.630	0.631	0.601	0.536

^1^Provided per kg of diet: 37.5 mg Zn (as ZnSO_4_), 37.5 mg of Mn (MnO_2_), 37.5 mg of Fe (as FeSO_4_•7H_2_O), 3.75 mg of Cu (as CuSO_4_•5H2O), 0.83 mg of I (as KI), and 0.23 mg of Se (as Na_2_SeO_3_•5H_2_O)

^2^Provided per kg of diet: 15,000 IU of vitamin A, 3,750 IU of vitamin D_3_, 37.5 mg of vitamin E, 2.55 mg of vitamin K_3_, 3 mg of thiamin, 7.5 mg of riboflavin, 4.5 mg of vitamin B_6_, 24 μg of vitamin B_12_, 51 mg of niacin, 1.5 mg of folic acid, 0.2 mg of biotin and 13.5 mg of pantothenic acid

##### Growth performance

The broiler chickens were weighed individually, and body weight (BW) was recorded at 14 d and 24 d to calculate the body weight gain (BWG), feed intake (FI), and feed conversion ratio (FCR). Mortality was recorded as it occurred.

##### Diarrhea and intestinal lesion score

After challenging coccidia and IBD vaccines, diarrhea score were measured from each cage (5 replicates/treatments) until final experimental period (9 d). Diarrhea score were evaluated following the method by Morehouse et al. [24]. Macroscopic intestinal coccidial lesions were scored in all birds at the end of the experiment using a method adopted in the study by Johnson and Reid [25]. After euthanizing broilers at the end of the experiment, the jejunum and ileum were scored separately. Score 0: normal, Score 1: contain some bloody spot and normal digesta, Score 2: serosal surface has red petechiae and contains some bloody spots, Score 3: contain large quantities of blood, caecal mould and mucus, Score 4: increased its thickness and contain bloody spots which have specific colour and smell.

### Exp. 2

#### Establishment of NE disease model

NE was induced according to the optimal conditions determined in Exp. 1. Briefly, birds in the challenged groups were orally gavaged × 20 the recommended dose of coccidia vaccine (Hipra Evalon®, Laboratorios Hipra, Girona, Spain) and IBD vaccine (IBD Blen®, Boeheringer Ingelheim Animal Health USA Inc., Georgia, USA) on 14 days of age followed by oral gavage with a 3 mL of CP type A NCTC 8798 (NCTC, National Collection of Type Cultures, London, UK) at 2.2 × 10^7^ CFU/mL twice daily for three consecutive days (09:00 and 17:00; on 18, 19, and 20 d). Uninfected control broilers received the same dosage of sterile phosphate-buffered saline by oral gavage.

#### Experimental animals and treatment

A 3 × 2 completely randomized factorial design was used to investigate the effects of supplementation of STB and multi-feed additives without and with NE challenge (NE-challenged or unchallenged). Seventy-two 1-day-old (initial body weight: 40.17 ± 0.27 g) Arbor Acres broilers were obtained from a local hatchery (Cherrybro Co., Asan, Korea). Broilers were randomly allocated into six experimental groups, and each experimental group had 4 replicate pens with three birds per pen. Dietary treatments were; non-additive supplemented control (CON), stimbiotic supplementation at 100 mg/kg (STB, Signis, AB Vista, UK), and stimbiotic combined with essential oil, probiotics, and yeast mannan (CB). The CB contained recommended dose of commercial symbiotics containing both bacterial strain of *Enterococcous*, *Pediococcus*, *Bifidobacterium* and *Lactobacillus* species and fructooligosaccharide as prebiotic (Poultry star® me, Biomin Holding GmbH, Austria), essential oils containing plant materials such as herbs, spices and essential oils characterized by mint oil, anise and clove (Digestarom® DC power, Biomin Holding GmbH, Austria) and prebiotics containing mannan-oligosaccharides and β-glucans (Safmannan®, Alltech, USA). The three diets were tested in broilers either non-infected or experimentally infected with the pre-established NE model. Broiler chickens were raised in the floor pens until 14 days of age and moved to battery cages on 14 d where they remained in the cage until 30 days of age. The environment of the room was maintained at 23 ± 1℃ of temperature and 50 ± 5% of relative humidity. Broiler chickens were offered antibiotic-free, coccidiostat-free and crumble diet according to the National Research Council (Table 1) [23] for the starter (1–7 d), grower (8–21 d) and finisher (22–30 d) periods. All broiler chickens are allowed to consume feed and water ad libitum*.*

#### Growth performance

The broiler chickens were weighed individually, and BW was recorded on d 1, 7, 14, 21 and 30. Birds were weighed individually but feed intake was measured per pen. BWG, FI, and FCR were calculated. Mortality was recorded as it occurred. Body weight-corrected FCR (bwcFCR) was calculated with an average BWG at 1659 g and 1 point of FCR being equivalent to 25 g on weight bain as the following formula:

bwcFCR = FCR – ((BWG period (g)—1659)/2500).

#### Organ characteristics

Twelve broilers from each treatment group were euthanized on 30 days of age by an intravenous injection of pentobarbital (100 mg/kg body weight), with cervical dislocation to confirm death. After broilers were euthanized, the abdomen was opened to excise and weigh the small intestine segments (duodenum, jejunum, and ileum), liver, spleen, and bursa of Fabricius. The weights of the small intestines segments and immune organs were recorded and the relative organ weight calculated using the following formula: Relative organ weight (g/kg) = organ weight (g)/live BW (kg). The length of the small intestine was recorded and the relative length calculated using the following formula: Relative intestinal length (cm/kg) = intestinal length (cm)/live BW (kg).

#### Footpad dermatitis and intestine lesion scores

Macroscopic footpad dermatitis and coccidial intestinal lesions were scored in all birds at the end of the experiment using a method used by Rushen et al. [26], and Johnson and Reid [25]. After euthanizing broilers, footpad lesions were scored on a scale from Score 0: no FPL, Score 1: the central part of the pad is raised, reticulate scales are separated, and small black necrotic areas may be present, Score 2: marked swelling of the footpad & less than one-quarter of the total area of the footpad, Score 3: swelling is evident & one half of the footpad, Score 4: more than half the footpad covered by necrotic cell. The lesion scores of jejunum and ileum were scored according to the standard referred to Exp. 1, separately.

#### Ileal morphology

Four broilers per treatment (one from each pen) were randomly selected and sacrificed at the end of the experiment to collect ileal tissue samples. A 15-cm ileal segment adjacent to the pyloric valve was freed of mesenteric attachments and rinsed with 10% neutral buffered formalin. The intestinal segment was submerged in approximately 20 mL of 10% neutral buffered formalin for 24 h. Slides of intestinal cross-Sects. (5 µm thick) were processed in low-melt paraffin and stained with hematoxylin and eosin. The stained slides were scanned by fluorescence microscopy (TE2000, Nikon, Tokyo, Japan) with a charge-coupled device (CCD) camera (DS-Fi1; Nikon, Tokyo, Japan) to measure intestinal morphology. The villus height (VH) was measured from the tip of the villus to the crypt orifice. Crypt depth (CD) was measured from the junction of the villus to the crypt base. And then, the villus height-to-crypt-depth ratio (VH:CD) was calculated.

#### Blood profile

Blood samples were collected from the brachial wing vein into a sterile syringe. At the time of collection, blood samples were collected into both no heparinized tubes and vacuum tubes containing tripotassium ethylene diamine tetra-acetic acid (K_3_EDTA; Becton, Dickinson and Co., Franklin Lakes, NJ, USA) to obtain serum and whole blood, respectively. The concentrations of lymphocytes in the whole blood samples were analyzed with an automatic biochemical analyzer (RA-1000, Bayer Corp., Tarrytown, NY, USA) using corresponding regent kits (Zhongsheng Biochemical Co., Ltd., Beijing, China). The immunoglobulin A (IgA), immunoglobulin G (IgG) and immunoglobulin M (IgM) concentrations in serum were measured using an automatic biochemistry analyzer (HITACHI 747; Boehringer Mannheim) following instructions of the commercial kits (Zhangsheng Biochemical Co., Ltd., Beijing, China). Tumor necrosis factor-alpha (TNF-α) and endotoxin were determined by a commercial ELISA kit (Nanjing Jiancheng Institute of Bioengineering). The measurement procedures were strictly in accordance with the protocols of the manufacturer.

#### Cecal microbiota

At the end of the experiment, the cecal digesta of one broiler from each pen (4 replications per treatment) was aseptically collected into individual sterile culture tubes and then placed on ice for transportation to the laboratory, where analysis was immediately carried out. One gram of cecal sample was blended with 9 mL of 1× PBS buffer and vortexed for 1 min. Counts of viable bacteria in the cecal samples were determined by plating tenfold serial dilutions (10^−1^ to 10^−8^) onto Lactobacilli MRS agar (MB cell, Seoul, Korea), MacConkey agar (MB cell, Seoul, Korea) and Perfringens tryptose-sulfite-cycloserine and Shahidi-Ferguson perfringens agar base (TSC and SFP; Oxoid, Ontario, Canada) mixed with egg yolk emulsion (Oxoid, Ontario, Canada) to isolate *Lactobacillus*, *Escherichia coli (E. coli)*, and *Clostridium perfringens*, respectively. The Lactobacilli and MacConkey agar plates were then incubated for 24 h at 37 °C under anaerobic conditions. The Perfringens agar plates were incubated for 24 h at 37 °C under anaerobic conditions. After the incubation periods, colonies of the respective bacteria were counted and expressed as the logarithm of colony-forming units per gram (log_10_ CFU/g).

### Statical analysis

Parametric data (growth performance, ileal morphology, blood profile and bacterial count in ceacum) were submitted to ANOVA using the Standard Least Squares model using JMP Pro 15.1 (SAS Institute Inc., Cary, NC, USA). The statistical model included the effect of CP challenge (No, Yes), the effect of vaccines challenge (0, × 10, × 20) and the interaction between CP and coccidia vaccine challgenge in Exp. 1, and the additives (CON, STB, CB), the effect of the challenge (No, Yes) and the interaction between additives and challenge in Exp. 2, and initial body weight at start of the trial (d0) was also included as a covariate. Treatment means were separated using Student’s *t*-test with significance accepted at *P* ≤ 0.05, with trends discussed when 0.05 < *P* ≤ 0.10. Non-parametric data (diarrhea, footpad dermatitis and intestine lesion scoring) were analysed using contingency analysis to test the relationship between categorical variables (scores) and the different combinations tested in this study. A Chi-square test was performed to determine if the different combinations had an effect on the categorical variables repartition with significance accepted at *P* ≤ 0.05.

## Results

### Exp. 1

#### Growth performance

There was no significant interaction (*P* > 0.05) between *Clostridium perfringens* (CP) infection and vaccine overdose in growth performance among treatments (Table 2). CP challenged birds decreased (*P* < 0.01) BW, BWG and FI compared with non-CP challenged birds on 24 d. Moreover, the injection of vaccine decreased (*P* < 0.05) BW, BWG and FI compared to the CON on 24 d. No mortality was recorded during the whole experimental period.

**Table 2:** Effects of vaccine overdose and *C.perfringens* infection on growth performance in broiler chickens (Exp. 1)^1^

*Clostridium* *perfringens* (C)	Vaccine (V)	BW		BWG	FI	FCR
		14 d	24 d			
No	0	381	910	530	940	1.78
No	× 10	378	891	511	909	1.78
No	× 20	382	898	518	914	1.77
Yes	0	375	897	518	907	1.75
Yes	× 10	383	866	487	870	1.79
Yes	× 20	379	866	487	855	1.74
SEM^2^		3.5	9.0	10.3	8.5	0.02
*Clostridium* *Perfringens*						
No		380	899^a^	520^a^	921^a^	1.78
Yes		379	877^b^	497^b^	877^b^	1.76
	Vaccine					
	0	378	903^a^	524^a^	923^a^	1.77
	× 10	380	878^b^	498^b^	890^b^	1.79
	× 20	381	882^b^	502^b^	88^b^	1.76
*P*-value						
C		0.95	0.003	0.003	< 0.001	0.66
V		0.98	0.01	0.01	0.001	0.80
C × V		0.99	0.58	0.58	0.39	0.11

Mortality was not observed in this experiment

*n* = 20 birds/treatment. All birds received allotted treatment diet starting study 0 d

^1^0, non-vaccine challenge; × 10, oral administration the levels of × 10 recommended dose of vaccine; × 20, oral administration the levels of × 20 recommended dose of vaccines; No, non-oral challenged with CP; Yes, oral challenged with 3 mL of 2.2 × 10^7^ CFU CP; *BW* body weight, *BWG* body weight gain, *FI* feed intake, *FCR* feed conversion ratio

^2^*SEM* standard error of mean

^a,b^Means in the same column with different superscripts are significantly different by Student’s test (*P* < 0.05)

#### Incidence of diarrhea and intestinal lesion

**Fig. 1:**
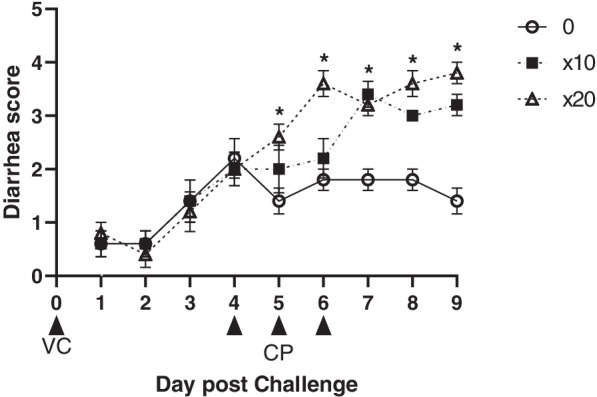
Effects of vaccine overdose and *C. perfringens* (CP) challenge on average diarrhea score in broilers for 9 d (Exp. 1). 0, non-vaccine challenge; × 10, oral administration the levels of × 10 recommended dose of vaccines; × 20, oral administration the levels of × 20 recommended dose of vaccines; CP: oral challenged with 3 mL of 2.2 × 10^7^ CFU CP. *n* = 5 pen/treatment.^*^Significant difference were observed among treatment (*P* < 0.05)

**Fig. 2:**
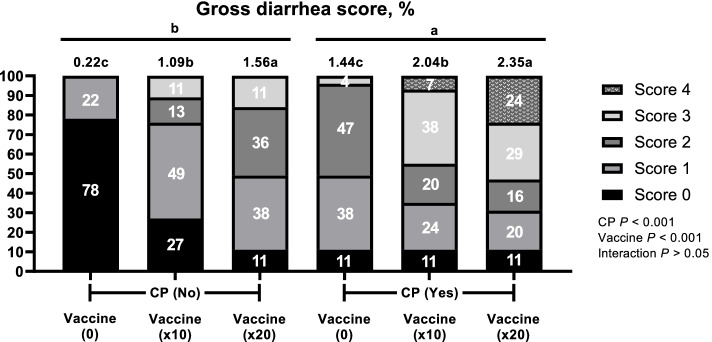
Effects of vaccine overdose and *C. perfringens* (CP) challenge on diarrhea score for 9 d in broilers (Exp. 1). 0, non-vaccine challenge; × 10, oral administration the levels of × 10 recommended dose of vaccine; × 20, oral administration the levels of × 20 recommended dose of vaccines; No, non-oral challenged with CP; Yes, oral challenged with 3 mL of 2.2 × 10^7^ CFU CP. *n* = 5 pen/treatment. χ^2^ = 173.283, *P* < 0.001. Numbers inside the bar indicates percentatge of score out of total (100%) as shown in legned. ^a,b,c^Means scores followed by different superscript in the bar graph indicates statistical significant by the Student’s test (*P* < 0.05)

**Fig. 3:**
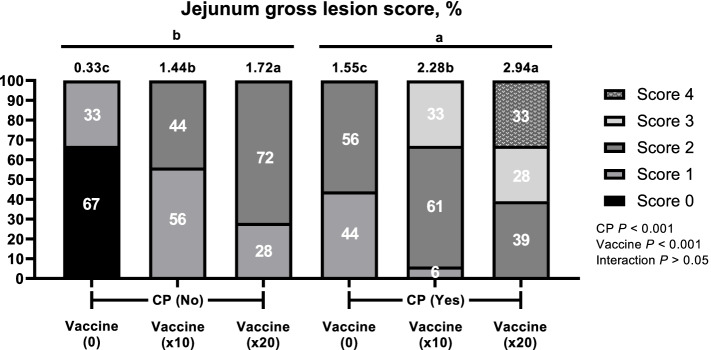
Effects of vaccine overdose and *C. perfringens* (CP) challenge on lesion score of jejunum in broilers (Exp. 1). 0, non-vaccine challenge; × 10, oral administration the levels of × 10 recommended dose of vaccine; × 20, oral administration the levels of × 20 recommended dose of vaccines; No, non-oral challenged with CP; Yes, oral challenged with 3 mL of 2.2 × 10^7^ CFU CP. *n* = 20 birds/treatment. χ^2^ = 140.065, *P* < 0.0001. Numbers inside the bar indicates percentatge of score out of total (100%) as shown in legned. ^a,b,c^Means scores followed by different superscript in the bar graph indicates statistical significant by the Student’s test (*P* < 0.05)

**Fig. 4:**
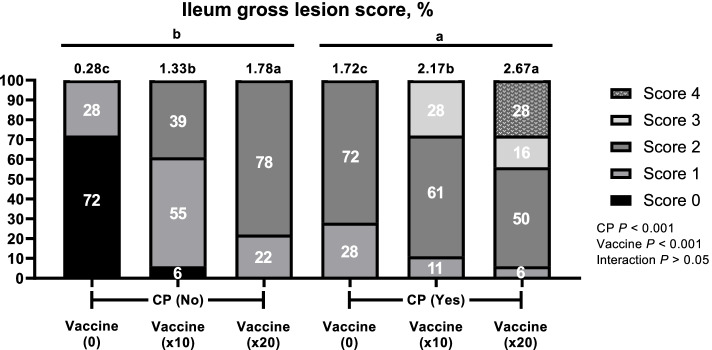
Effects of vaccine overdose and *C. perfringens* (CP) challenge on lesion score of ileum in broilers (Exp. 1). 0, non-vaccine challenge; × 10, oral administration the levels of × 10 recommended dose of vaccine; × 20, oral administration the levels of × 20 recommended dose of vaccines; No, non-oral challenged with CP; Yes, oral challenged with 3 mL of 2.2 × 10^7^ CFU CP. *n* = 20 birds/treatment. χ^2^ = 126.802, *P* < 0.0001. Numbers inside the bar indicates percentatge of score out of total (100%) as shown in legned. ^a,b,c^Means scores followed by different superscript in the bar graph indicates statistical significant by the Student’s test (*P* < 0.05)

After coccidi and IBD challenge, there were no significant difference (*P* > 0.05) on average diarrhea scores from 1 to 4 d and then broilers challenged with × 20 vaccines overdose and CP had highest (*P* < 0.05) average diarrhea scores compared with boilers challenged with 0 and × 10 vaccines and CP (Fig. 1). An overview of the prevalence and scores of the diarrhea and intestinal lesion scores with and without CP and vaccine overdose challenge is shown in Fig. 2, 3 and 4. CP and vaccine overdose challenges significantly increased (*P* < 0.001) incidence of higher diarrhea, and intestinal lesion scores in the jejunum and ileum increased as the harshness of the challenges were increased. As the level of vaccine overdose increased, both diarrhea and intestinal lesion scores were increased (*P* < 0.001). Especially, the highest prevalence of diarrhea and intestinal lesion scores were observed in birds exposed to the × 20 vaccine overdose along with CP infection, closely mimicking the commercial NE outbreak.

### Exp. 2

#### Growth performance

There was no significant interactions (*P* > 0.05) between treatments and NE challenge in growth performance (Table 3 and 4). There were no significant differences (*P* > 0.05) in BW, BWG, FI, FCR and bwcFCR between treatments during the pre-challenge period (from 1 to 14 d, Table 3 and 4). After the NE challenge, BW, BWG and FI were decreased (*P* < 0.05) compared with non-NE challenged groups until the end of the experiment (14 to 21 d). The NE challenge increased (*P* < 0.001) FCR only for a short-term between 14 to 21 d and contributed to increased (*P* < 0.001) FCR and bwcFCR between 1 to 30 d. The birds fed either STB or CB diet had a heavier (*P* < 0.05) BW on 21 d and 30 d compared with the birds fed a CON diet, but there was no significant BW difference (*P* > 0.05) between birds fed STB and CB diet. The birds fed either STB or CB diet improved (*P* < 0.05) BWG, FCR and bwcFCR in 14–21 d and 1–30 d compared with the birds fed a CON diet, but there were no differences (*P* > 0.05) between birds fed STB and CB diet. The dietary treatment did not alter (*P* > 0.05) feed intake in both the NE-challenged and non-challenged groups. No mortality was recorded during the entire experimental period.

**Table 3:** Effects of dietary treatments and NE challenge on growth performance in broilers (Exp. 2)^1^

Treatment (T)	Challenge (C)	BW, g					BWG, g				
		1 d	7 d	14 d	21 d	30 d	1–7 d	7–14 d	14–21 d	21–30 d	1–30 d
CON	NE-	40	185	446	867	1,714	145	261	421	847	1,674
STB	NE-	40	187	451	897	1,780	147	264	446	883	1,740
CB	NE-	40	189	455	900	1,798	149	265	446	897	1,757
CON	NE +	40	184	445	779	1,583	144	261	333	805	1,543
STB	NE +	40	188	451	805	1,678	148	263	354	873	1,638
CB	NE +	40	189	453	819	1,670	148	264	366	851	1,629
SEM^2^			2.5	4.8	11.8	32.6	2.5	4.8	11.4	32.6	32.6
Treatment											
CON		40	184	445	823^b^	1,648b	144	261	377^b^	826	1,608^b^
STB		40	188	451	851^a^	1,729a	147	263	400^a^	878	1,689^a^
CB		40	189	454	860^a^	1,734a	149	265	406^a^	874	1,693^a^
	Challenge										
	NE-	40	187	450	888^a^	1,764a	147	263	438^a^	876	1,724^a^
	NE+	40	187	449	801^b^	1,644^b^	147	263	351^b^	843	1,603^b^
*P*-value											
T		-	0.19	0.22	0.007	0.02	0.19	0.72	0.034	0.21	0.02
C		-	0.86	0.81	< 0.001	< 0.001	0.86	0.87	< 0.001	0.22	< 0.001
T × C		-	0.92	0.97	0.89	0.89	0.92	0.98	0.84	0.83	0.89

Mortality was not observed in this experiment

*n* = 16 birds/treatment. All birds received allotted treatment diet starting study 0 d

^1^*CON*, no feed additives; *STB*, 100 mg/kg Signis from 0–30 d; *CB*, 100 mg/kg Signis, and 500 mg/kg Poultry star, 150 mg/kg Digestarom and 500 mg/kg Saf-mannan from 0–7, 7–21 and 21–30 d, respectively; *NE-*, non NE challenge; *NE+* , NE challenge

^2^*SEM* standard error of means

^a,b^Means in the same column with different superscripts are significantly different by Student’s test (*P* < 0.05)

**Table 4:** Effects of dietary treatments and NE challenge on growth performance in broilers (Exp. 2) ^1^

Treatment (T)	Challenge (C)	FI, g					FCR					bwc FCR
		1–7 d	7–14 d	14–21 d	21–30 d	1–30 d	1–7 d	7–14 d	14–21 d	21–30 d	1–30 d	
CON	NE-	160	344	665	1,536	2,705	1.11	1.32	1.59	1.87	1.63	1.62
STB	NE-	163	357	653	1,555	2,727	1.12	1.36	1.47	1.78	1.57	1.54
CB	NE-	161	346	663	1,564	2,734	1.08	1.31	1.50	1.77	1.56	1.52
CON	NE +	159	351	600	1,470	2,579	1.11	1.35	1.74	1.86	1.68	1.72
STB	NE +	159	349	598	1,492	2,598	1.08	1.33	1.71	1.84	1.63	1.66
CB	NE +	165	355	598	1,496	2,615	1.11	1.35	1.65	1.77	1.61	1.62
SEM^2^		1.9	8.0	7.9	49.9	51.9	0.017	0.036	0.037	0.065	0.002	0.028
Treatment												
CON		159	347	632	1,503	2,642	1.11	1.33	1.66	1.86	1.65^a^	1.67^a^
STB		161	353	625	1,523	2,663	1.10	1.34	1.59	1.81	1.60^ab^	1.60^b^
CB		163	351	631	1,530	2,675	1.10	1.33	1.57	1.77	1.58^b^	1.57^b^
	Challenge											
	NE-	161	349	660^a^	1,551	2,722^a^	1.10	1.33	1.^52b^	1.81	1.59^b^	1.56^b^
	NE+	161	352	599^b^	1,486	2,597^b^	1.10	1.34	1.70^a^	1.82	1.64^a^	1.67^a^
*P*-value												
T		0.19	0.80	0.65	0.85	0.82	0.85	0.96	0.08	0.41	0.05	0.006
C		0.80	0.66	< 0.001	0.13	0.01	0.77	0.72	< 0.001	0.75	0.01	< 0.001
T × C		0.12	0.51	0.77	0.99	0.99	0.13	0.61	0.46	0.82	0.86	0.91

*n* = 16 birds/treatment. All birds received allotted treatment diet starting study 0 d

^1^*CON*, no feed additives; *STB*, 100 mg/kg Signis from 0–30 d; *CB*, 100 mg/kg Signis, and 500 mg/kg Poultry star, 150 mg/kg Digestarom and 500 mg/kg Saf-mannan from 0–7, 7–21 and 21–30 d, respectively; *NE-*, non NE challenge; *NE+* , NE challenge

^2^*SEM* standard error of mean

^a,b^Means in the same column with different superscripts are significantly different by Student’s test (*P* < 0.05)

#### Organ characteristic

There was no interactions (*P* > 0.05) between treatment and NE challenge in relative intestinal length and weight (Table 5). The NE challenge increased (*P* < 0.05) the relative weight of the liver and spleen while decreasing (*P* < 0.05) the relative weight of the bursa of Fabricius compared with the non-challenged group. There was no dietary treatment effect (*P* > 0.05) on the organ characteristics.

**Table 5:** Effects of dietary treatments and NE challenge on organ characteristics in broilers (Exp. 2)^1^

Treatment (T)	Challenge (C)	Relative intestinal length, cm/kg of live BW			Relative intestinal weight, g/kg of live BW			Relative immune organ weight, g/kg of live BW		
		Duodenum	Jejunum	Ileum	Duodenum	Jejunum	Ileum	Liver	Spleen	Bursa of Fabricius
CON	NE-	19.0	38.6	39.2	6.4	10.3	6.6	21.5	0.80	2.54
STB	NE-	19.4	36.6	40.4	6.3	10.2	6.8	21.5	0.81	2.54
CB	NE-	16.5	39.2	40.0	6.5	10.2	6.6	21.2	0.95	2.54
CON	NE+	17.4	36.6	40.3	6.2	10.0	6.8	21.8	1.09	1.66
STB	NE+	17.5	38.1	39.7	6.1	9.9	6.7	23.3	1.03	1.85
CB	NE+	19.5	36.6	40.1	6.4	10.1	6.6	23.5	1.01	1.87
SEM^2^		0.32	0.48	0.45	0.19	0.37	0.32	0.76	0.061	0.134
Treatment										
CON		18.2	37.6	39.75	6.28	10.16	6.68	21.65	0.94	2.10
STB		18.5	37.3	40.04	6.23	10.03	6.73	22.41	0.92	2.19
CB		18.0	37.9	40.03	6.43	10.10	6.64	22.33	0.98	2.20
	Challenge									
	NE-	18.3	38.1	39.86	6.39	10.19	6.67	21.40^b^	0.85^b^	2.54^a^
	NE+	18.1	37.1	40.01	6.24	10.00	6.70	22.86^a^	1.04^a^	1.79^b^
*P*-value										
T		0.85	0.93	0.83	0.58	0.94	0.97	0.39	0.20	0.67
C		0.46	0.95	0.73	0.33	0.53	0.90	0.02	< 0.001	< 0.001
T × C		0.23	0.19	0.82	0.99	0.98	0.92	0.55	0.66	0.68

*n* = 16 birds/treatment. All birds received allotted treatment diet starting study 0 d

^1^*CON*, no feed additives; *STB*, 100 mg/kg Signis from 0–30 d; *CB*, 100 mg/kg Signis, and 500 mg/kg Poultry star, 150 mg/kg Digestarom and 500 mg/kg Saf-mannan from 0–7, 7–21 and 21–30 d, respectively; NE-, non NE challenge; NE+ , NE challenge

^2^*SEM* standard error of mean

^a,b^Means in the same column with different superscripts are significantly different by Student’s test (*P* < 0.05)

#### Footpad dermatitis and intestine lesion

**Fig. 5:**
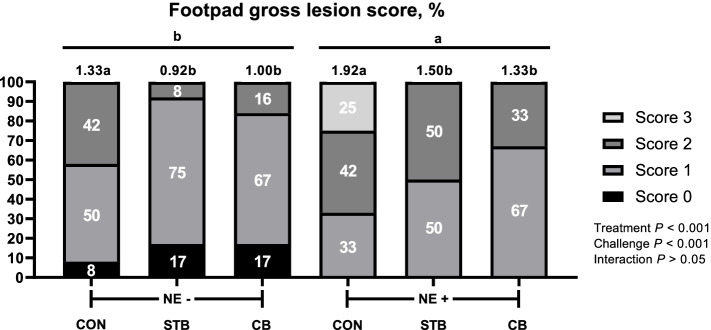
Effects of dietary treatments and NE challenge on footpad dermatitis in broilers (Exp. 2). CON, no feed additives; STB, 100 mg/kg Signis from 0–30 d; CB, 100 mg/kg Signis, and 500 mg/kg Poultry star, 150 mg/kg Digestarom and 500 mg/kg Saf-mannan from 0–7, 7–21 and 21–30 d, respectively; NE-, non NE challenge; NE+ , NE challenge. *n* = 16 birds/treatment. χ^2^ = 28.176, *P* < 0.05. Numbers inside the bar indicates percentatge of score out of total (100%) as shown in legned. ^a,b^Means scores followed by different superscript in the bar graph indicates statistical significant by the Student’s test (*P* < 0.05)

**Fig. 6:**
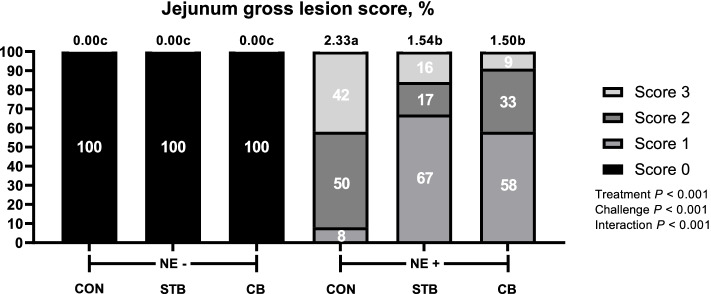
Effects of dietary treatments and NE challenge on lesion scores of jejunum in broilers (Exp. 2). CON, no feed additives; STB, 100 mg/kg Signis from 0–30 d; CB, 100 mg/kg Signis, and 500 mg/kg Poultry star, 150 mg/kg Digestarom and 500 mg/kg Saf-mannan from 0–7, 7–21 and 21–30 d, respectively; NE-, non NE challenge; NE + , NE challenge. *n* = 16 birds/treatment. χ^2^ = 93.250, *P* < 0.0001. Numbers inside the bar indicates percentatge of score out of total (100%) as shown in legned. ^a,b,c^Means scores followed by different superscript in the bar graph indicates statistical significant by the Student’s test (*P* < 0.05)

**Fig. 7:**
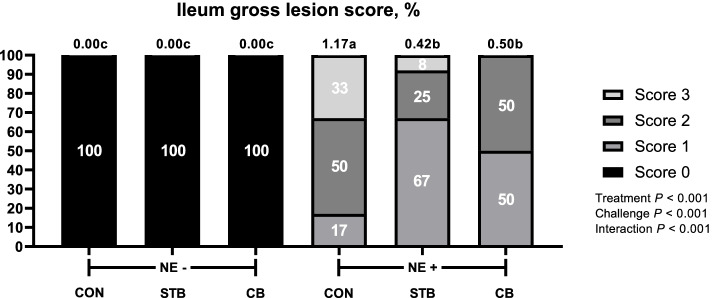
Effects of dietary treatments and NE challenge on lesion scores of ileum in broilers (Exp. 2). CON, no feed additives; STB, 100 mg/kg Signis from 0–30 d; CB, 100 mg/kg Signis, and 500 mg/kg Poultry star, 150 mg/kg Digestarom and 500 mg/kg Saf-mannan from 0–7, 7–21 and 21–30 d, respectively; NE-, non NE challenge; NE+ , NE challenge. *n* = 16 birds/treatment. χ^2^ = 42.646, *P* < 0.0001. Numbers inside the bar indicates percentatge of score out of total (100%) as shown in legned. ^a,b,c^Means scores followed by different superscript in the bar graph indicates statistical significant by the Student’s test (*P* < 0.05)

The results of the incidence of footpad dermatitis and intestinal lesion score are shown in Figs. 5, 6 and 7. Broilers supplemented STB and CB with NE challenge or non NE challenge showed the incidence of lower footpad dermatitis scores (*P* < 0.05) and intestinal lesion score (*P* < 0.0001; *P* < 0.0001) compared with control group. The NE challenge increased footpad dermatitis scores (*P* < 0.001) compared to non-challenged broilers. The supplementation of STB and CB reduced footpad dermatitis (*P* < 0.001) compared with control group. There were significant interaction (*P* < 0.001) among treatments and challenge. Broilers supplemented STB and CB with NE challenge had lower average intestinal lesion scores compared with those of broiler fed CON with NE challenge.

#### Ileal morphology

There were no interactive effects (*P* > 0.05) of dietary treatment and NE challenge on ileal villus height (VH), crypt depth (CD) and VH:CD ratio (Table 6). The NE challenge decreased (*P* < 0.001) VH and increased (*P* < 0.001) CD, hence reducing (*P* < 0.001) VH:CD ratio. The supplementation of STB and CB resulted in improved VH and VH:CD ratio (*P* < 0.001) compared with CON broilers, but there was no significant difference (*P* > 0.05) between STB and CB.

**Table 6:** Effects of dietary treatment and NE challenge on ileal morphology in broilers (Exp. 2)^1^

Treatment (T)	Challenge (C)	Villus height, µm	Crypt depth, µm	VH:CD
CON	NE-	1206	104	12.5
STB	NE-	1291	94	15.7
CB	NE-	1360	93	15.3
CON	NE+	1006	124	8.4
STB	NE+	1213	111	12.0
CB	NE+	1201	113	10.8
SEM^2^		32.7	6.8	0.95
Treatment				
CON		1106^b^	114	10.4^b^
STB		1252^a^	102	13.8^a^
CB		1280^a^	103	13.1^a^
	Challenge			
	NE-	1286^a^	97^b^	14.5^a^
	NE+	1140^b^	116^a^	10.4^b^
*P*-value				
T		< 0.001	0.17	0.002
C		< 0.001	< 0.001	< 0.001
T × C		0.18	0.96	0.93

*n* = 16 birds/treatment. All birds received allotted treatment diet starting study 0 d

^1^*CON*, no feed additives; *STB*, 100 mg/kg Signis from 0–30 d; *CB*, 100 mg/kg Signis, and 500 mg/kg Poultry star, 150 mg/kg Digestarom and 500 mg/kg Saf-mannan from 0–7, 7–21 and 21–30 d, respectively; *NE-*, non NE challenge; *NE+* , NE challenge

^2^*SEM* standard error of mean

^a,b^Means in the same column with different superscripts are significantly different by Student’s test (*P* < 0.05)

#### Blood profile

**Fig. 8:**
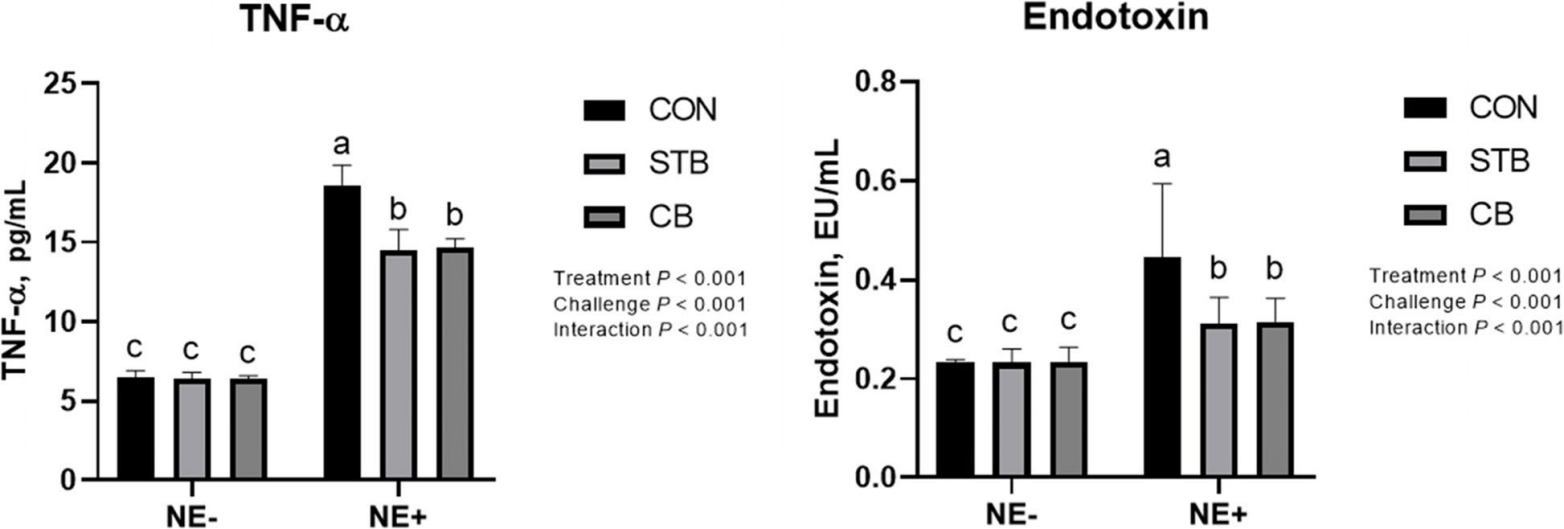
Effects of dietary treatments and NE challenge on *TNF-α* and endotoxin level in broilers (Exp. 2). CON, no feed additives; STB, 100 mg/kg Signis from 0–30 d; CB, 100 mg/kg Signis, and 500 mg/kg Poultry star, 150 mg/kg Digestarom and 500 mg/kg Saf-mannan from 0–7, 7–21 and 21–30 d, respectively; NE-, non NE challenge; NE+ , NE challenge. *n* = 4 birds/treatment. ^a,b,c^Means scores followed by different superscript in the bar graph indicates statistical significant by the Student’s test (*P* < 0.05)

There were no significant difference and interaction on lymphocyte among dietary treatment and NE challenge (Table 7). The NE-challenge significantly decreased (*P* < 0.05) serum IgG and increased (*P* < 0.001) serum endotoxin and *TNF-α* contents (Fig. 8). There was a significant treatment by NE-challenge interaction in serum endotoxin and *TNF-α* content (*P* < 0.001) such that supplementation of STB and CB decreased serum *TNF-α* and endotoxin content only in challenged birds. However, there were no differences (*P* > 0.05) in serum endotoxin and *TNF-α* content between STB and CB.

**Table 7:** Effects of dietary treatment and NE challenge on blood profile in broilers (Exp. 2)^1^

Treatment (T)	Challenge (C)	Lymphocyte, %	IgA, mg/dL	IgG, mg/dL	IgM, mg/dL
CON	NE-	71.3	2.00	4.75	2.00
STB	NE-	71.0	1.25	4.75	1.75
CB	NE-	72.6	1.75	4.25	1.75
CON	NE +	72.4	1.25	2.00	1.25
STB	NE +	72.7	1.25	3.75	1.25
CB	NE +	72.5	1.50	3.75	1.50
SEM^2^		1.44	0.332	0.663	0.326
Treatment					
CON		71.9	1.63	3.38	1.63
STB		71.8	1.63	4.25	1.50
CB		72.6	1.50	4.00	1.63
	Challenge				
	NE-	71.6	1.83	4.58^a^	1.83
	NE+	72.5	1.33	3.17^b^	1.33
*P*-value					
T		0.85	0.91	0.42	0.92
C		0.44	0.08	0.02	0.08
T × C		0.83	0.76	0.23	0.76

*n* = 4 birds/treatment. All birds received allotted treatment diet starting study 0 d

^1^*CON*, no feed additives; *STB*, 100 mg/kg Signis from 0–30 d; *CB*, 100 mg/kg Signis, and 500 mg/kg Poultry star, 150 mg/kg Digestarom and 500 mg/kg Saf-mannan from 0–7, 7–21 and 21–30 d, respectively; *NE-*, non NE challenge; *NE+* , NE challenge

^2^*SEM* standard error of mean

^a,b^Means in the same column with different superscripts are significantly different by Student’s test (*P* < 0.05)

#### Cecal microbiota

**Fig. 9:**
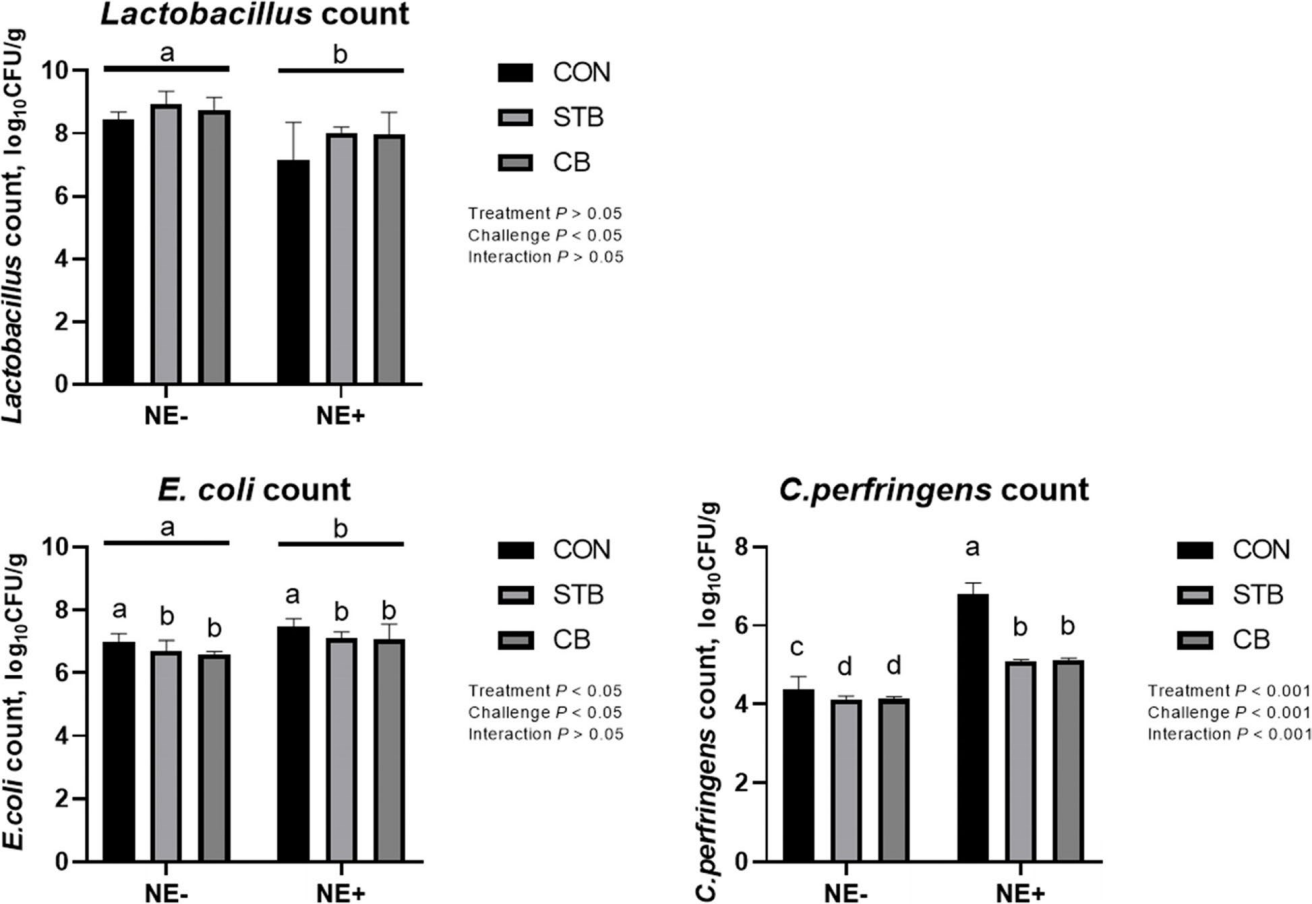
Effects of dietary treatments and NE challenge on *C. perfringens* count in caecal content of broilers (Exp. 2). CON, no feed additives; STB, 100 mg/kg Signis from 0–30 d; CB, 100 mg/kg Signis, and 500 mg/kg Poultry star, 150 mg/kg Digestarom and 500 mg/kg Saf-mannan from 0–7, 7–21 and 21–30 d, respectively; NE-, non NE challenge; NE+ , NE challenge. *n* = 4 birds/treatment. ^a,b,c^Means scores followed by different superscript in the bar graph indicates statistical significant by the Student’s test (*P* < 0.05)

The NE challenge significantly reduced (*P* < 0.001) *Lactobacillus* count in the cecal content and increased (*P* < 0.001) *E. coli* and *C. perfringens* counts (Fig. 9). Supplementation of either the STB or CB did not affect (*P* > 0.05) *Lactobacillus* count, while they simultaneously significantly decreased(*P* < 0.001) *E. coli* and *C. perfringens* counts. There was treatment by NE-challenge interaction (*P* < 0.001) on *C. perfringens* count, such that supplementation of STB and CB significantly reduced *C. perfringens* count in cecal digesta in both non-challenged and challenged birds but more extensively in challenged birds.

## Discussion

The ban on the use of antimicrobials in livestock diets have caused the re-emergence of NE accompanied by increased mortality rate and economic losses to the poultry industry [7]. To be able to test the efficacy of a functional additive under the condition of commercial NE outbreak, experimental induction of NE has been used in many research facilities. For the establishment of the NE model, many studies have been used the co-infection methods with CP and *Eimeria* spp. [27–30]. Also, these studies have evaluated the gut health and growth performance based on the feed efficiency in broiler chickens challenged with NE [3, 31–35]. The studies examined alternative methods to replace antibiotics through the use of bacteriophage, essential oils, synbiotics (probiotics and prebiotics), enzymes, organic acids and antimicrobial peptides. However, the lack of reproducibility of the NE disease model has been a major hindrance in estimating the effects of dietary treatments to alleviate typical clinical symptoms of NE [27]. Previous studies reported that the mortality caused by NE in established models varied from 0 to 60% [36–38]. To establish a more robust NE infection model, we examined the extent of the vaccine overdose to suppress the immune response prior to a series of oral doses of CP. Unlike previous studies which used 10 times cocci + IBD vaccine overdose through the ocular route prior to CP inoculation [39], we experimentally induced NE with oral vaccine overdose challenges which is a more effective route of infection for *Eimeria* spp and CP. As hypothesised, the 20 times vaccine overdose triggered more severe diarrhoea scores and intestinal lesion scores compared to the birds with no vaccine treatment or 10 times vaccine overdose.

The overdose of vaccine with CP infection decreased growth performance including final BW, BWG and FI, and caused higher diarrhea and intestinal lesion scores compared against control broilers. This result was consistent with the results reported by Song et al. [40] and Wu et al. [41] whereby NE challenges decreased growth performance and resulted in poorer morphology of the small intestine and increased intestinal lesion score. Skinner et al. [42] reported that experimental induction of NE increased FCR by 11% and decreased BW by 12%. Also, consistent with our finding, coccidial infection disrupted the intestinal barrier, thereby allowing the penetration of pathogenic CP into the mucosal membrane which exacerbated the severity of the intestinal lesion scores [43–45]. In particular, the challenge group using vaccines at × 20 dose with CP inoculation decreased growth performance and increased the incidence of severe diarrhea and intestinal lesion score compared with other groups including vaccines at × 10 dose with CP inoculation and non-challenge.

In this study, we did not observe mortality with the NE challenge. This result was inconsistent with the results of Caly et al. [46] who reported increased mortality following the NE challenge. These contradictions may be explained by the differences in challenge period, type and dosage of vaccine, and the genotype of broilers. Although no birds were dead in this experiment, challenge with coccidial and IBD vaccine and CP dramatically increased the loss of BW and decreased the feed intake. Also,NE challenge increased the incidence of severe diarrhea and intestinal lesion scores, and reduced FI and BWG in broilers. Based on the results, high overdose vaccines with CP challenge successfully caused severe diarrhea and intestinal lesion scores and reduce growth performance. Therefore, we used immunosuppression via oral overdose of 20 times cocci and IBD vaccines and a series of oral CP challenges (3 mL of 2.2 × 10^7^ CFU/mL) as a model for experimental induction of NE in Exp. 2.

In the present Exp. 2, NE challenged boilers had higher relative liver and spleen weight, the incidence of severe footpad dermatitis score, but lower bursa of Fabricius weight and VH and VH/CD ratio than non-NE challenged birds. Also, NE challenge increased pathogenic bacterial counts (*E. coli* and *C. perfringens*) and decreased *Lactobacillus* count and growth performance. These results were consistent with the previous studies which showed that wet litter (> 30 per cent moisture) is associated with increased incidence and severity of FPD in broilers [47]. *Enteritis* often causes diarrhea, resulting in increased nutrient and moisture excretion into the litter [48]. The dual action of *Eimeria* spp. is to provide a suitable environment for CP infection and subsequent physical epithelial invasion by the virulence factors [37, 49], and *Eimeria* challenge reduced growth performance and intestinal VH [50]. Liu et al. [51] showed that the NE challenge promoted the proliferation of several gram-negative bacteria in the ileum, such as *E. coli*, resulting in endotoxin translocation to the blood and increasing the endotoxin level in the blood. Collectively, the experimental induction of NE was successful in Exp. 2 and therefore assures the validity of testing the second and third hypotheses.

The second hypothesis tested in Exp. 2 was that STB supplementation will partly reduce the extent of the performance loss and the inflammatory response triggered by the NE challenge. Corn occupied most of poultry diets contain considerable levels of arabinoxylans as part of non-starch polysaccharides. XOS is more likely to perform as an activator of specific bacteria in the gastrointestinal tract (GIT) than directly act as a quantitative prebiotic, thereby increasing SCFA including butyrate can be helpful to gut health [18, 19]. The stimbiotic used in this study was designed to convey XOS to fibrolytic microflora in the large intestine by adding a small portion of ex-vivo produced short-chain XOS with a β-1,4-endo xylanase [20], which hydrolyzes the arabinoxylan portion in the feed to XOS in the GIT. In this experiment, STB supplementation partly ameliorated the loss of growth performance and reduced inflammatory response induced by the NE challenge. In the case of body weight gain and FCR, STB comparably recovered the performance of the NE challenged birds to the performance of the non-challenged control birds. On the other hand, observation on the translocation of bacterial lipopolysaccharides into the circulation system (serum endotoxin concentration) and innate immune response to the increased endotoxin circulation (serum TNF-α concentration) clearly showed the interactive effect of STB, where STB partly reduced endotoxin translocation and innate immune response in the NE challenged birds.

The integrity of the intestinal barrier is compromised by coccidial infection, which damages the intestinal mucosal layer, thereby enabling the penetration of pathogenic CP into the mucosal membrane to promote the onset of severe NE lesions [43, 45]. Our study showed that the addition of Stimbiotics (STB and CB) in NE challenged birds prevented the mucosal damage measured by villous height and crypt depth. It was reported that the provision of XOS can improve intestinal morphology, structure and intestinal microflora population [52, 53]. The VH and VH/CD ratio are the common criteria for estimating the nutrient absorption capacity of the small intestine, and a higher VH and VH/CD ratio means a higher absorptive capacity of the small intestine [53, 54]. The present study also found that STB supplementation increased beneficial microbial count while reducing the pathogenic bacterial counts. These results were in agreement with the study of Ding et al. [53] and Teng et al. [50], who reported that XOS improved intestinal microflora, thereby positively affecting the relative length of the small intestine, VH, and VH/CD ratio of the jejunum of laying hens. Consistent with these findings, previous studies also showed that supplementation with 2 or 5 g/kg XOS in the diet increased the proportion of the genus *Lactobacillus* in cecal concentrations [53–56]. Moreover, XOS supplementation had significantly improved the relative abundances of bacteria often considered beneficial such as *Coprococcus*, *Lactobacillus*, *Roseburia*, and *Ruminococcus* in addition to increased luminal concentrations of SCFAs. Supplementation of XOS perhaps provides a receptor for Gram-negative pathogens on the intestinal surface, whereby XOS can serve as the attachment site for Gram-negative pathogens promoted by *C. perfringens* and prevent attachment of bacteria onto the enterocytes [57, 58].

The final hypothesis tested in Exp. 2 was that supplementation of additional additives to that of STB will show a limited additional beneficial effect in broilers challenged with NE. Supplementation of probiotics, essential oils and yeast mannan that acts as pseudo-mucosal glycoprotein receptor and binding to pathogens, on top of STB supplementation did not improve growth performance and reduce the inflammatory response under the experimental condition compared with STB alone. This is probably due to the additional additives overlapping with the STB with regards to mode of action. For example, probiotics, prebiotics and essential oils all target the intestinal microbiome by either supplementing beneficial bacteria, supplementing nutrients for the proliferation of beneficial bacteria or controlling the growth of the pathogenic bacteria. Eventually, all of these additives would have contributed to the proliferation of beneficial bacteria while limiting the growth of the pathogens. Interestingly, under the experimental condition used in Exp. 2, STB supplementation alone was sufficient enough to reduce the severity of the NE inflammation. Although that was true in this particular experimental condition, this may not mean that probiotics, essential oils and yeast mannan are not effective. If birds are experiencing multiple inflammatory and environmental challenges which are often common in the commercial production system, those additives may work additively or synergistically depending on the type of and the severity of the challenge.

## Conclusions

This experiment successfully established a NE infection model in broilers. The NE challenge decreased growth performance and increased indices of inflammatory response. STB supplementation, which delivered specific xylooligosaccharides to the intestinal microbiome improved the growth performance and partly reduced the inflammatory response of the NE challenged birds. Further supplementation of probiotics, essential oils and yeast mannan on top of STB supplementation did not improve performance and the inflammatory response over and above the STB alone under the experimental condition.

## Data Availability

The datasets used and analysed during the current study are available from the corresponding author on reasonable request.

## Abbreviations

STB: Stimbiotic
NE: Necrotic enteritis
CP: Clostridium perfringens
BW: Body weight
BWG: Body weight gain
FI: Feed intake
FCR: Feed conversion ratio
bwcFCR: Body weight-corrected FCR
VH: Villus height
CD: Crypt depth
IgA: Immunoglobulin A
IgG: Immunoglobulin G
IgM: Immunoglobulin M
TNF-α: Tumour necrosis factor-alpha
XOS: Xylo-oligosaccharides
EX: Exogenous xylanase
SCFA: Short-chain fatty acids
GIT: Gastrointestinal tract
CFU: Colony forming unit
