# Correction: Stimbiotic supplementation modulated intestinal infammatory response and improved broilers performance in an experimentally-induced necrotic enteritis infection model

**DOI:** 10.1186/s40104-022-00797-x

**Published:** 2022-10-31

**Authors:** Ji Hwan Lee, Byongkon Lee, Xavière Rousseau, Gilson A. Gomes, Han Jin Oh, Yong Ju Kim, Se Yeon Chang, Jae Woo An, Young Bin Go, Dong Cheol Song, Hyun Ah Cho, Jin Ho Cho

**Affiliations:** 1 Department of Animal Science, Chungbuk National University, Cheongju, 28644 South Korea; 2 Cherrybro Co., Ltd., Jincheon-Gun, 27820 South Korea; 3 AB Vista, Marlborough, Wiltshire UK

**Correction: J Anim Sci Biotechnol 13, 100 (2022)**

**https://doi.org/10.1186/s40104-022-00753-9**

Following publication of the original article [1], the authors reported three typos in the title and the text.

The original title is “Stimbiotic supplementation modulated intestinal infammatory response and improved boilers performance in an experimentally-induced necrotic enteritis infection model”, in which “broilers” was mistakenly spelled as “boilers”.

The correct title should read: “Stimbiotic supplementation modulated intestinal infammatory response and improved broilers performance in an experimentally-induced necrotic enteritis infection model”.

In section “Incidence of diarrhea and intestinal lesion” on page 5, the word “boilers” was mistakenly spelled and should be changed to “broilers”.

On page 12, the word “boilers” in “In the present Exp. 2, NE challenged boilers had higher relative liver and spleen weight, …” was mistakenly spelled and should be changed to “broilers”.

The original article [1] has been updated.
